# Enhanced Detection of Acute Ischemic Stroke With Low-Field MRI

**DOI:** 10.1161/SVIN.125.002110

**Published:** 2026-01-21

**Authors:** Annabel Sorby-Adams, Nandor K. Pinter, Amelia Demopoulos, John Kirsch, Vinay Jaikumar, Olivia K. Nelson, Stephen Bacchi, Jennifer Guo, Blair A. Parry, Hailey Brigger, Ian Johnson, Adam de Havenon, Gordon Sze, Rafael O’Halloran, John Pitts, Vivien H. Lee, Keith W. Muir, Shahid M. Nimjee, Adnan Siddiqui, Kathryn E. Keenan, Matthew S. Rosen, Juan Eugenio Iglesias, Kevin N. Sheth, Joshua N. Goldstein, W. Taylor Kimberly

**Affiliations:** Department of Neurology and the Center for Genomic Medicine (A.S.-A., A.D., S.B., J.G., W.T.K.), Massachusetts General Hospital and Harvard Medical School, Boston.; Department of Emergency Medicine (O.K.N., B.A.P., J.N.G.), Massachusetts General Hospital and Harvard Medical School, Boston.; Department of Radiology, Athinoula A. Martinos Center for Biomedical Imaging (J.K., M.S.R., J.E.I.), Massachusetts General Hospital and Harvard Medical School, Boston.; Department of Radiology (N.K.P.), Jacobs School of Medicine & Biomedical Sciences, State University of New York at Buffalo, NY.; Department of Neurosurgery (N.K.P., V.J., A.S.), Jacobs School of Medicine & Biomedical Sciences, State University of New York at Buffalo, NY.; Department of Neurology, Center for Brain & Mind Health, Yale New Haven Hospital and Yale School of Medicine, CT (H.B., I.J., A.d.H., K.N.S.).; Division of Neuroradiology, Department of Radiology and Biomedical Imaging, Yale New Haven Hospital and Yale University School of Medicine, CT (G.S.).; Hyperfine Incorporated, Guilford, CT (R.O.H., J.P.).; Department of Neurology (V.H.L.), The Ohio State University Wexner Medical Center, Columbus.; Department of Neurological Surgery (S.M.N.), The Ohio State University Wexner Medical Center, Columbus.; School of Psychology and Neuroscience, University of Glasgow, United Kingdom (K.W.M.).; National Institute of Standards and Technology, Boulder, CO (K.E.K.).; Hawkes Institute, University College London, United Kingdom (J.E.I.).; Department of Electrical Engineering and Computer Science, Computer Science and Artificial Intelligence Laboratory, Massachusetts Institute of Technology, Cambridge (J.E.I.).

**Keywords:** ischemic stroke, magnetic resonance imaging, neuroimaging, patients, signal-to-noise ratio

## Abstract

**BACKGROUND:**

Portable, low-field magnetic resonance imaging (MRI) has the potential to expand access to neuroimaging in environments where conventional MRI is limited. However, diffusion-weighted imaging at low magnetic field is challenged by a low signal-to-noise ratio and gradient strength, which may limit diagnostic confidence in acute ischemic stroke evaluation, particularly for very small strokes. In this study, we evaluated a combination of novel pulse sequences and low-field MRI hardware to enhance lesion detection.

**METHODS:**

Patients with a suspected diagnosis of acute ischemic stroke were prospectively enrolled at 3 centers. Diffusion-weighted imaging was performed using a single-direction (SD) or a custom multi-direction (MD) sequence comprising 3 orthogonal directions. Imaging was acquired on 2 0.064 T hardware versions: a first-generation C-arm system (Swoop v1) and a next-generation H-arm system featuring optimized gradient amplifiers, form factor, and cooling system (Swoop v2). Diagnostic accuracy and the lower limit of lesion detection were calculated for both SD and MD images on each system compared with ground-truth MRI (1.5–3 T).

**RESULTS:**

A total of 95 patients (n=62 confirmed acute ischemic stroke; n=33 stroke mimics) were included. On SD images, agreement between assessors regarding lesion detection was *κ*=0.72, and *κ*=0.84 on MD images. The positive predictive value for differentiating acute ischemic stroke from stroke mimics was 78.2% on SD and 95% on MD images. For SD images, a lesion volume cut point of 0.6 mL yielded a sensitivity of 89% and specificity of 88%. For MD images, the lesion volume cut point was 0.4 mL, with a corresponding sensitivity of 86% and specificity of 83%. MD imaging on the next-generation v2 system improved image uniformity (*P*<0.05), reduced scan time by ≈30%, and enabled the detection of lesions as small as 0.15 mL (2.8 mm maximum diameter).

**CONCLUSIONS:**

Implementation of diffusion-weighted imaging optimization strategies on low-field MRI improves detection of very small strokes in a clinically feasible time frame.

**CLINICAL PERSPECTIVE**

**What Is New?**

- Portable, low-field magnetic resonance imaging has the potential to increase access to neuroimaging for patients with acute ischemic stroke, yet it has limitations in sensitivity and signal-to-noise.
- In this study, we optimized low-field diffusion-weighted imaging/apparent diffusion coefficient pulse sequences and magnetic resonance imaging hardware to improve the detection of very small acute ischemic stroke lesions.

**What Are the Clinical Implications?**

- Incorporating low-field magnetic resonance imaging in acute stroke workflows has implications for the evaluation of minor stroke and transient ischemic attack, and potentially in resource-limited or prehospital settings.

Recent advances in low-field magnetic resonance imaging (LF-MRI) have the potential to improve access to MRI and facilitate the diagnosis and management of acute ischemic stroke (AIS). These devices operate at very low magnetic field strengths, enabling scanning outside the controlled environment of a shielded MRI suite, circumventing the need for liquid cryogens, reducing power requirements, and enabling portability.^1^ This simplification of operation and maintenance has the potential to enhance access in settings unable to support conventional MRI, including in emergency departments, intensive care units, and outpatient settings.^2–4^ MRI availability at the point-of-care may facilitate the detection of stroke mimics, which are a common presentation in emergency departments with a prevalence ranging from 20% to 50%,^5–7^ yet can be difficult to distinguish from AIS in the absence of advanced imaging.^8^

Diffusion-weighted imaging (DWI) is highly sensitive to AIS, where restricted diffusion, characterized by the compartmental shift of water from the interstitial to intracellular space, can be detected on DWI within minutes of stroke onset.^9^ Conversely, T2 fluid-attenuated inversion recovery (FLAIR) is sensitive to the movement of water from the vascular to the interstitial space, as seen in the delayed setting of ionic and vasogenic edema.^10^ The principles underlying FLAIR and DWI enable the differentiation of hyperacute versus subacute stroke^11–13^ and can guide subsequent patient management in unwitnessed or wake-up stroke.^14–16^ We previously showed that DWI at LF is possible,^3,17^ and in combination with FLAIR,^18^ can be used as a tissue clock similar to conventional MRI. However, limitations in LF-DWI detection of very small lesions may compromise clinical accuracy.

In this study, we sought to improve the sensitivity of LF-DWI for AIS by focusing on 2 core optimization strategies to enhance lesion detection: signal uniformity and image resolution. Currently available DWI on LF-MRI utilizes a single diffusion direction, whereas conventional MRI generates an average DWI map (trace-weighted image) from multiple diffusion directions, which improves sensitivity, signal-to-noise ratio, and reduces the confounding appearance of white matter tracts due to the minimization of fractional anisotropy, improving signal uniformity. To implement multi-direction (MD) DWI at LF, we developed an accelerated pulse sequence that enables 3-direction diffusion acquisition in a reduced timeframe. To further improve acquisition, we implemented a 3-direction DWI sequence on a next-generation 0.064 T scanner, which incorporates gradient amplifiers with higher power and a cooling system to mitigate thermal effects on the B_0_ field. To establish the clinical significance of these advances, we evaluated the sensitivity and specificity of ischemic lesion detection, comparing both pulse sequences and hardware systems.

## Methods

### Data Availability

The data generated and analyzed in the present study are available from the corresponding author on reasonable request.

### Study Design

This was a prospective multicenter observational study performed at Massachusetts General Hospital, Yale New Haven Hospital, and Buffalo General Medical Center. The primary objective of the study was to determine whether MD diffusion imaging improves differentiation of AIS from stroke mimics compared with single-direction (SD) acquisitions, and whether this effect is further enhanced with next-generation hardware systems. The primary outcome was diagnostic accuracy, defined as the ability of MD relative to SD DWI to correctly classify AIS versus stroke mimics, quantified using receiver operating characteristic analysis against 1.5–3 T MRI acquired for clinical care.

Patients with suspected AIS were enrolled between June 2023 and January 2025. Exclusion criteria included evidence of intracranial hemorrhage on admission, noncontrast computed tomography, age <18 years, pregnancy, electrically stimulated implants such as cardiac pacemakers, and a body weight exceeding 400 lbs (181.4 kg). All imaging was acquired for research purposes under an institutional review board–approved protocol from the respective institution, with informed consent obtained prospectively from patients or their legally authorized representative.

### Sequence Development and Image Acquisition

LF-MRI operating at a field strength of 0.064 T was used for all image acquisition. Between June 2023 and August 2024, LF-MRI imaging was performed on a US Food and Drug Administration–cleared, Swoop v1 clinical system (Mk1.9, Hyperfine, Inc) at each of the participating sites.^17^ On v1 hardware, the standard DWI protocol acquires a *b*=900 s/mm^2^ sequence in a single diffusion direction along the *z* axis (here referred to as SD), which corresponds to the anterior-posterior direction in the coordinate frame of the scanner accompanied by a single *b*=0 to derive an apparent diffusion coefficient (ADC) map (see Table 1 for full image acquisition parameters, including scan durations).

**Table 1.**
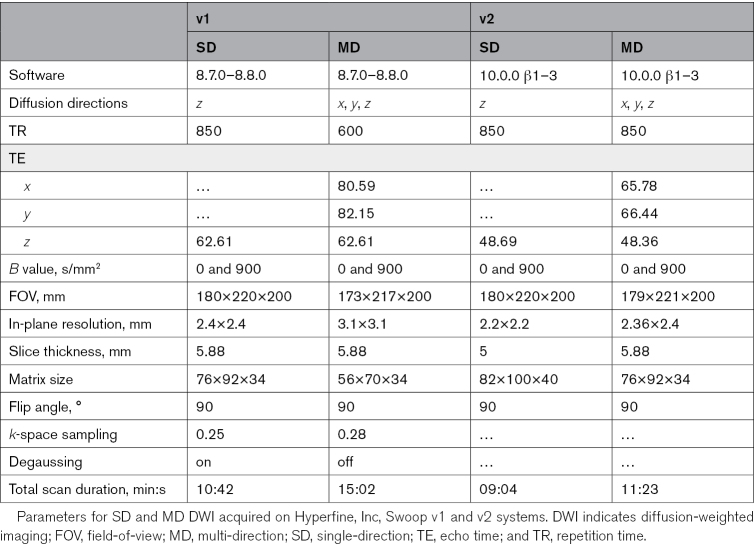
Sequence Parameters

We developed a custom, accelerated MD DWI sequence to enable sequential acquisition of 3 *b*=900 s/mm^2^ diffusion directions in orthogonal *x* (inferior to superior), *y* (left to right) and *z* (anterior to posterior) directions in the coordinate frame of the scanner (corresponding to *z*, *x* and *y* coordinates respectively in Talairach space), accompanied by a single *b*=0 (see Table 1 for full acquisition parameters). To generate trace DWI and ADC maps from the MD images, *b*=0 and b=900 s/mm^2^ *x*, *y*, and *z* diffusion directions were coregistered using a 12-parameter affine registration in NiftyReg^19^ with trace and ADC maps generated thereafter in MATLAB (v2024b, Natick, MA). Images and maps were then interpolated to 1 mm isotropic resolution in FreeSurfer^20^ (v7.4.2) for final interpretation.

Between May 2024 and January 2025, imaging was performed on a preproduction, next-generation system (hardware specification Swoop v2, Hyperfine, Inc, US Food and Drug Administration–cleared June 2025) at the Massachusetts General Hospital and Yale New Haven Hospital. This hardware version incorporates several design modifications that include a wider form factor, reduced B_0_ inhomogeneity, gradient amplifiers with increased power, and a cooling system for the gradient coils. These modifications were implemented to optimize DWI and ADC imaging and enable acquisition in a shorter duration than v1 counterparts and thus improve clinical utility (Table 1). The v2 system supports both SD and MD (*x*, *y*, *z*) DWI (*b*=900 s/mm^2^). For the MD sequence, trace and ADC maps were calculated directly on the scanner without the need for offline postprocessing.

All patients underwent LF-MRI acquisition using either the v1 or v2 system in their hospital bed or stretcher in the emergency departments or inpatient units. Imaging was performed by trained research staff with the assistance of the bedside nurse. All patients underwent SD DWI, MD, or both, and T2 FLAIR acquisition. LF-MRI was obtained either before, after, or in the absence of 1.5 or 3 T MRI acquired for standard of care, with LF and available conventional imaging acquired within the same acute stroke period. Specifications for the v1 and v2 scanners are provided in Table S1.

### Imaging Interpretation

Two independent raters with >10 years of experience reading acute stroke images and familiarity with reading LF-MRI evaluated all images (N.K.P. and W.T.K.). Ground-truth diagnosis (AIS or stroke mimic) from conventional MRI acquired as standard of care was performed by consensus determination. LF SD and MD sequences from all patients were scored for the presence of DWI bright and ADC dark lesions. Interrater agreement was calculated between assessors for the detection of lesions >1 mL, as this represented the size that was reliably present on SD images. Assessment was repeated 6 weeks thereafter to determine intrarater agreement. Final LF-MRI assignments were resolved by consensus to enable comparison to ground-truth diagnoses on conventional neuroimaging. Lesions were also segmented from conventional DWI images using semiautomated thresholding in ITK-SNAP (v4.0.1)^21,22^ to determine the volume of each lesion.

### Postprocessing Pipeline

To evaluate the variability of the signal on SD and MD sequences and between v1 and v2 systems, a postprocessing pipeline was used to automatically segment sequences into whole brain, white matter, cortical gray matter, and deep gray matter (comprising the caudate, putamen, pallidum, and thalamus) regions-of-interest using machine learning pipelines SynthSR^23^ and SynthSeg^24,25^ deployed in FreeSurfer^20^ (v7.4.2). To optimize region segmentation, corresponding T2 FLAIR images were coregistered to SD or MD counterparts using a 12-parameter affine registration in NiftyReg,^19^ after which they underwent contrast synthesis and super-resolution (SynthSR) and segmentation (SynthSeg). Segmentation quality control scores were automatically estimated by SynthSeg (predictions of Dice scores for several brain regions made by a neural network). Images with automated segmentation quality control scores >0.7 across supratentorial regions and >0.1 across infratentorial regions were included for subsequent analysis, on which they were resampled to native DWI or ADC space. Visible stroke lesions on the corresponding SD or MD images were segmented using semiautomated thresholding as previously described, and the lesion was subtracted from the region of interest. Final regions were then applied to the SD and MD images and their associated ADC maps and the standard deviation of the signal extrapolated using MATLAB (v2024b, Natick, MA).

### Statistical Analysis

Statistical analyses were conducted in STATA (v18.0, StataCorp, College Station, TX) and RStudio (v2024.09.0, Boston, MA). Descriptive statistics were used to summarize cohort characteristics. Interrater and intrarater agreement on the v1 system was assessed using the Fleiss *κ* coefficient with 500 random sample bootstrap estimates. *κ* values were interpreted as follows: <0=poor, 0 to 0.20=slight, 0.21 to 0.40=fair, 0.41 to 0.60=moderate, 0.61 to 0.80=substantial, and 0.81 to 1.0=almost perfect.^26^ Diagnostic performance of the v1 and v2 systems was evaluated using positive predictive values, negative predictive values, sensitivity, and specificity. Receiver operating characteristic curves and corresponding area under the curve values were calculated to determine optimal lesion volume cut points. Logistic regression was performed to assess whether time from last known well to LF-MRI or time from LF to conventional MRI acquisition influenced the accuracy of lesion detection. Differences in signal variability were assessed using the Wilcoxon matched-pairs signed-rank test. Results are reported as 95% CIs and interquartile ranges. A *P*<0.05 was considered statistically significant.

## Results

### Patient Cohort

A total of 110 patients with stroke and stroke-like symptoms were enrolled. Of these patients, n=5 did not complete image acquisition due to clinical deterioration after consent (n=4) or incompatible body habitus (n=1). Of those who completed LF image acquisition, n=3 were excluded due to significant motion artifact precluding image analysis, and an additional n=7 patients were excluded due to the lack of conventional MRI for comparison. The final study cohort comprised n=95 individuals, including n=62 (65%) patients with imaging-confirmed AIS and n=33 (34%) with stroke mimics. Of these patients, n=67 underwent SD acquisition, n=8 underwent MD, and n=20 underwent both. Details of the study cohort by diagnosis on conventional neuroimaging are reported in Table 2, including baseline demographics, the system used for image acquisition, and imaging characteristics.

**Table 2.**
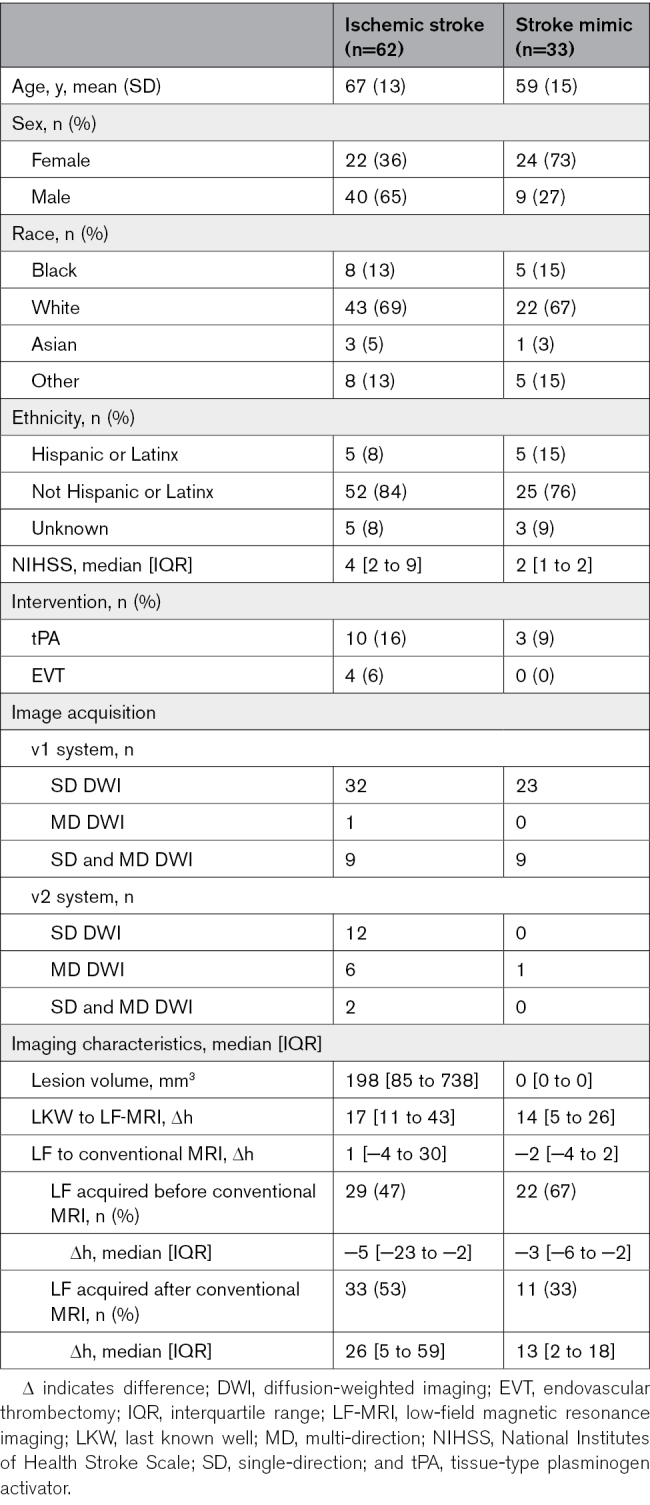
Cohort Demographics

**Figure 1.**
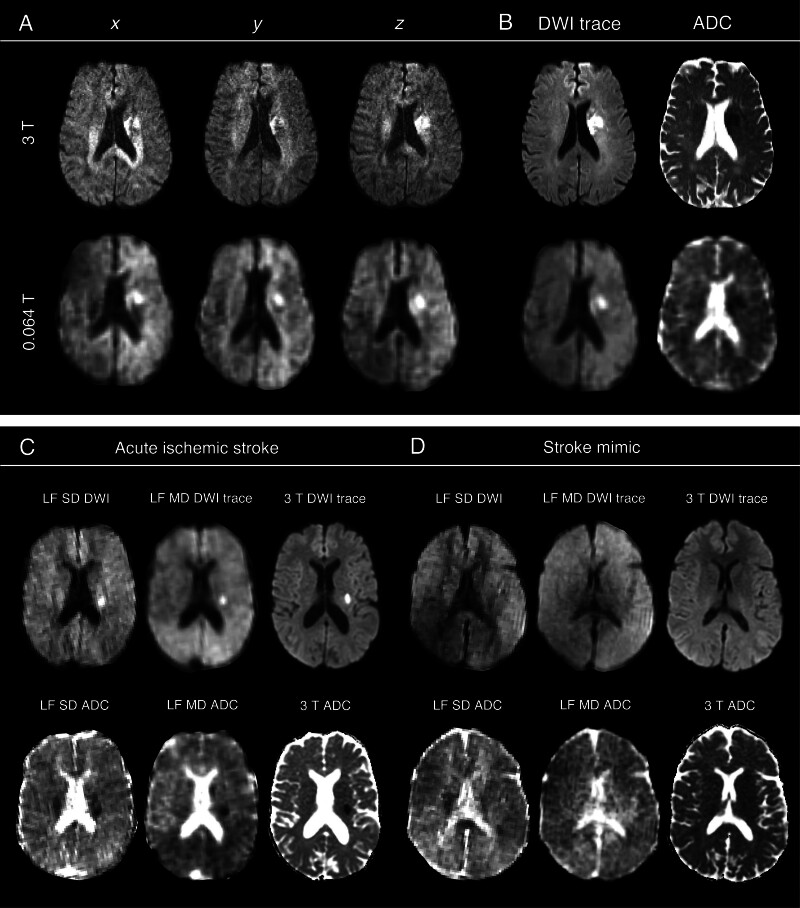
**Comparison of individual diffusion directions at 0.064 T (v1 system) and 3 T field strengths against averaged trace diffusion-weighted imaging (DWI) and apparent diffusion coefficient (ADC) counterparts. A**, Individual diffusion directions (*x*, *y*, and *z*) demonstrate variable lesion conspicuity at both field strengths. **B**, In comparison, averaged trace DWI and ADC counterparts show improved signal uniformity, whereby signal from anisotropically restricted white matter tracts is attenuated, enabling improved detection of isotropically restricted diffusion in ischemic cells. **C**, A case example from a patient with an acute ischemic lesion in the left corona radiata, which can more readily be seen on the 0.064 T multi-direction (MD) ADC map compared with the single-direction (SD), where corticospinal tracts obfuscate the lesion. **D**, A case example from a patient with a stroke mimic, where greater signal variability is seen on the SD 0.064 T DWI and ADC compared with MD counterparts. No diffusion abnormality can be seen on the conventional 3 T DWI nor ADC. The skull has been stripped for visualization purposes. Each image shown at 0.064 T is from v1 hardware. LF indicates low-field.

An example of LF SD and MD DWI and ADC images acquired on the v1 system, when compared with corresponding conventional MRI, is shown in Figure 1. Figure 1A shows the individual diffusion directions at 0.064 T and 3 T, and Figure 1B shows the resulting DWI trace and ADC maps. A second example of a small infarct in the left corona radiata is shown in Figure 1C, where the ADC signal is obscured by the normal restricted diffusion of white matter tracts in the SD but is detectable on the averaged ADC map. For comparison, a stroke mimic is shown in Figure 1D.

### Intrarater and Interrater Agreement

We first sought to evaluate the agreement between assessors regarding their ability to identify ischemic lesions on the v1 system. Overall, assessors were in moderate agreement for both SD and MD counterparts. Interrater agreement was *κ*=0.72 (95% CI, 0.56–0.88) on SD. This increased on MD, with an agreement of *κ*=0.84 (95% CI, 0.62–1.0). On repeat assessment, the intrarater agreement on SD was *κ*=0.75 (95% CI, 0.56–0.92), while agreement on MD was *κ*=0.84 (95% CI, 0.16–1.0).

### Sensitivity and Limit of Detection for Ischemic Lesions

**Figure 2.**
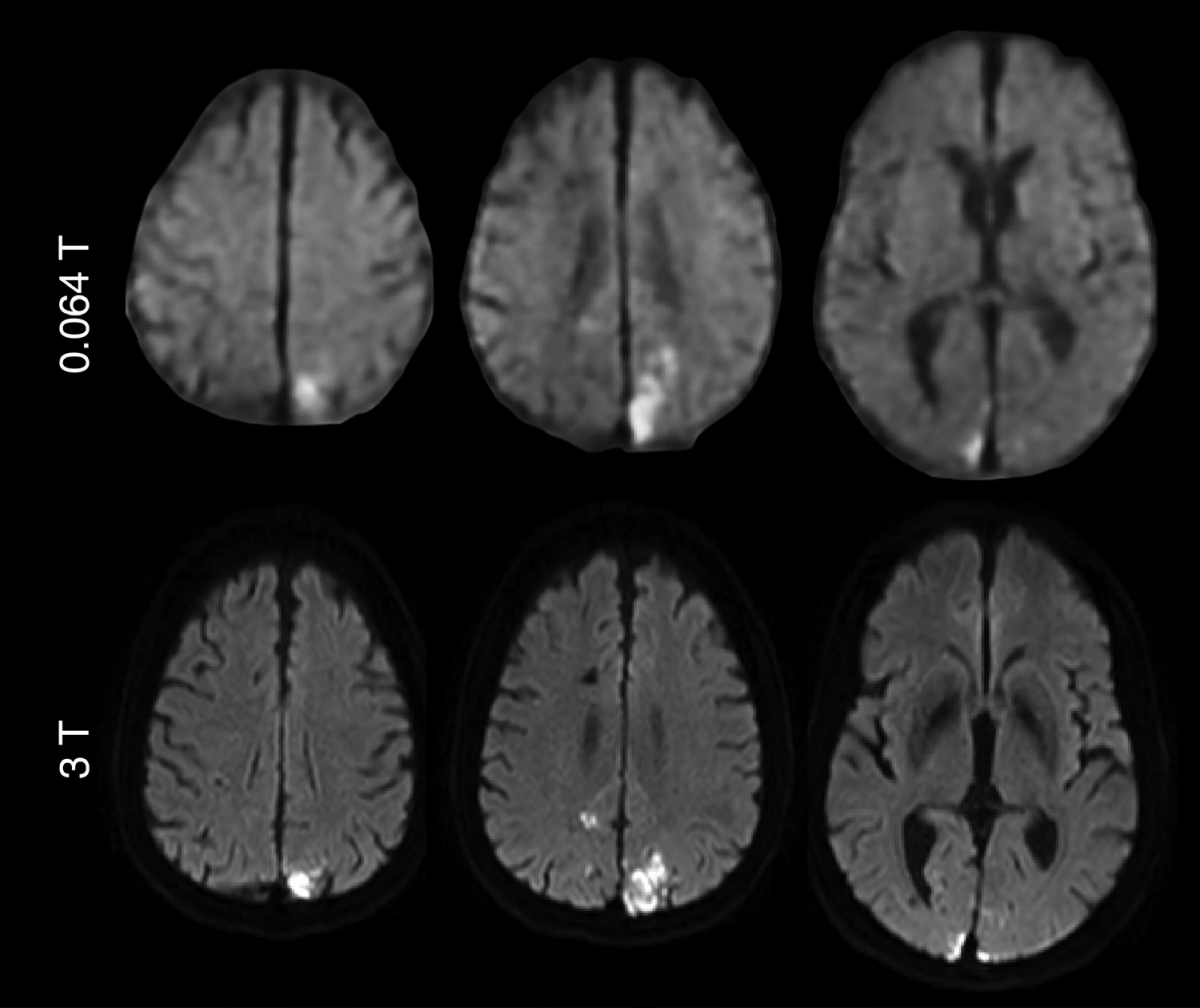
**Averaged trace diffusion-weighted imaging (DWI) on the next-generation v2 system.** Comparison between low-field trace DWI on the v2 system against conventional 3 T counterparts in a patient with bilateral acute ischemic infarcts in the occipital lobe. The skull has been stripped for visualization purposes. Each image shown at 0.064 T is from v2 hardware.

We next sought to determine the performance of the MD and SD sequences on the v1 and v2 systems relative to ground-truth conventional imaging. An example of images generated on the v2 system is shown in Figure 2, where MD images are shown relative to conventional MRI.

Diagnostic performance was first evaluated to determine the accuracy of differentiating AIS from stroke mimics on SD and MD counterparts, where a majority of the stroke mimics were collected on the v1 system. On SD images, the positive predictive value was 97.7% (95% CI, 88.3–99.9) and the negative predictive value was 71.4% (95% CI, 55.4–84.3), with a sensitivity of 78.2% (95% CI, 65–88.2) and specificity of 96.8% (95% CI, 83.3–99.9). In comparison, MD images were associated with a positive predictive value of 100% (95% CI, 82.4–100), negative predictive value of 90.9% (95% CI, 58.7–99.8), and sensitivity of 95% (95% CI, 71.5–99.9) and specificity of 100% (95% CI, 69.2–100).

**Figure 3.**
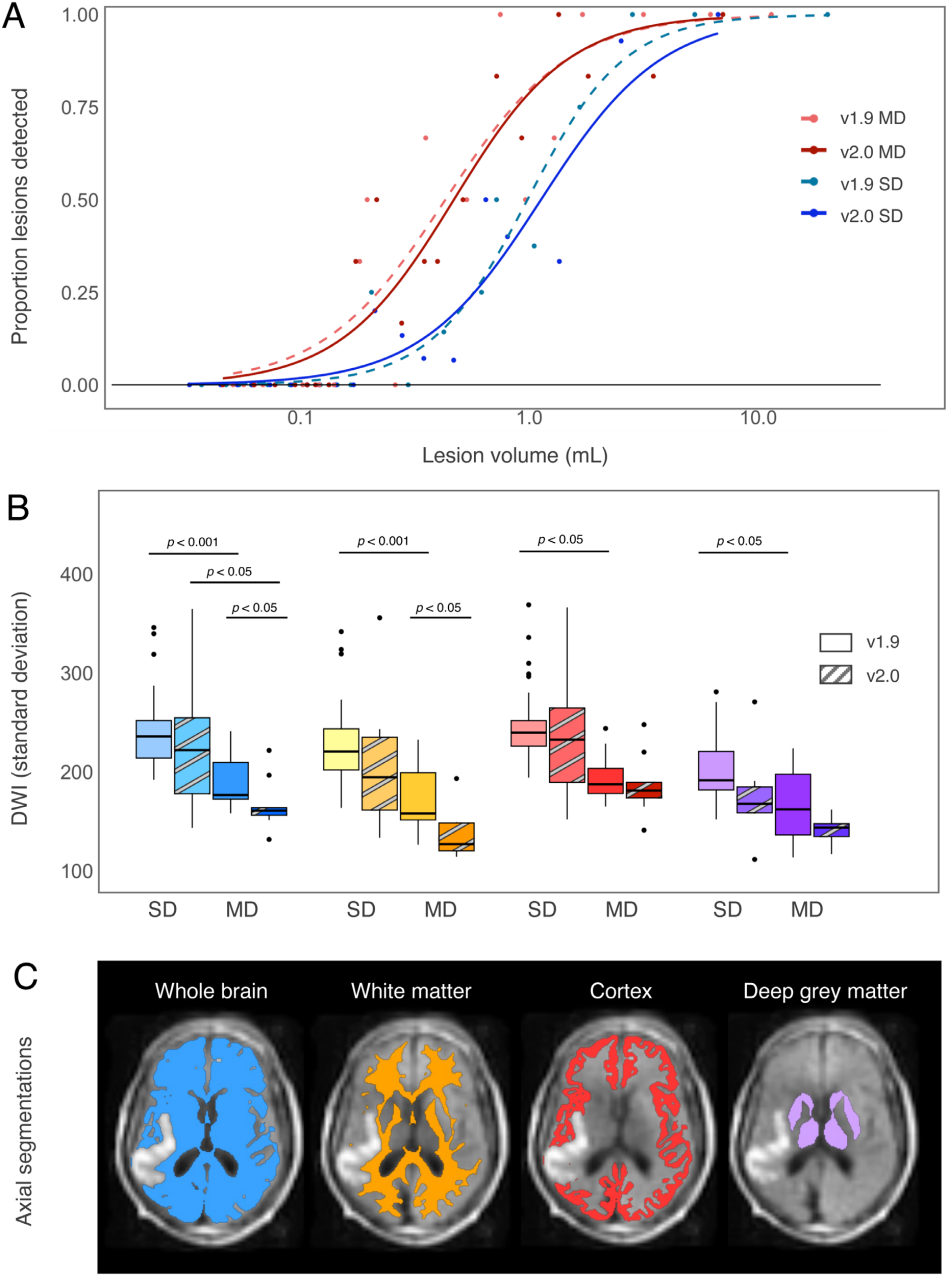
**Comparison of optimization strategies for enhanced lesion detection on v1 and v2 low-field systems. A**, The proportion of lesions correctly detected relative to lesion size. **B**, Signal standard deviation from single-direction (SD) and multi-direction (MD) images by scanner and anatomic region, including the whole brain (blue), white matter (orange), cortex (red), and deep gray matter (purple). **C**, Visual representation of anatomic segmentation regions from which the signal standard deviation is derived, less the lesion.

Next, we evaluated the sensitivity and specificity of detecting individual acute lesions in those with AIS. Lesions as small as 0.15 mL could be detected on the MD images on the v2 system and 0.19 mL on the v1 system. In comparison, the smallest detectable lesions on the SD images were 0.20 mL for both the v2 and v1 systems. Receiver operating characteristic analyses were subsequently performed to evaluate the lesion volume on both SD and MD to determine the optimal sensitivity and specificity for detection. For SD sequences, the lesion detection cut point was 0.6 mL with a sensitivity of 89% and specificity of 88%, and an area under the curve of 0.94 (95% CI, 0.92–0.97). For MD sequences, the lesion detection cut point value was 0.4 mL, with a corresponding sensitivity of 86% and specificity of 83% and an area under the curve of 0.93 (95% CI, 0.88–0.97). For lesions > 1.0 mL, detection on SD was associated with a sensitivity of 70% (95% CI, 57.7–80.7) and specificity of 82.8% (95% CI, 77.6–87.1), while on MD there was 100% sensitivity and specificity (95% CI, 95.9–100 and 91.8–100, respectively). Between the v1 and v2 systems, sensitivity was comparable (*P*>0.05; Figure 3A); however, there was a reduction in acquisition time by ≈30% using the v2 system.

The time from last known well to LF image acquisition did not influence the sensitivity or specificity of detection, irrespective of sequence or system (all *P*>0.05), nor did the time between LF and conventional MR acquisition (all *P*>0.05).

### Image Characteristics

Finally, we evaluated the uniformity of the signal in different regions (whole brain, white matter, cortex, and deep gray matter) for SD and MD images on both scanners (Figure 3B, 3C). A significant improvement in signal uniformity (ie, a reduction in inhomogeneity) was observed in the white matter on MD images compared with SD counterparts (*P*<0.05 for both v1 and v2 scanners). Similar differences were observed in the whole brain, cortex, and deep gray matter on the v1 scanner, where the MD was more uniform than SD (*P*<0.05). The MD sequence on the v2 system was more uniform than the v1 system in both the white matter and whole brain (*P*<0.05).

## Discussion

In this study, we optimized DWI for enhanced detection of AIS on point-of-care, LF-MRI. Acquisition in multiple diffusion directions and generation of averaged trace DWI and ADC maps increased both the specificity and sensitivity for detecting AIS, enabling accurate differentiation from stroke mimics. Moreover, the next-generation v2 scanner demonstrated improved signal uniformity, similar or slightly better detection of very small ischemic lesions, and reduced image acquisition time.

The role and type of imaging evaluation in acute stroke have undergone rapid change, especially in large vessel occlusion patients who may be candidates for thrombectomy. However, a majority of patients with ischemic stroke present with mild neurological deficits or with transient symptoms. In these patients, LF-MRI offers a potential tool for timely diagnosis and evaluation. For example, imaging biomarkers have been shown to improve patient prognostication and risk stratification when incorporated with clinical scores such as the ABCD2.^27–29^ Although our prior work has shown that DWI is feasible at low field strength,^17,18^ sensitivity to very small lesions remains limited. To address this gap, we implemented and evaluated sequential optimization strategies to improve lesion detection by enhancing signal uniformity and image resolution, evaluating images acquired in patients with mild stroke and stroke mimics.

We first developed an accelerated 3-direction trace DWI and ADC map to improve image uniformity and enhance lesion detection on existing (v1) LF-MRI hardware. MD trace images enabled the accurate differentiation of stroke from stroke mimics, with higher diagnostic agreement to conventional imaging than SD images and improved interrater and intrarater agreement. In keeping with these findings, the MD DWI eliminated the anatomic restricted diffusion signal associated with directional white matter tracts (eg, the corticospinal tracts). Averaging of the 3 diffusion directions for the trace DWI improved signal uniformity in these regions, attenuating signal related to fibers in any SD, improving lesion detection, particularly of those residing within or adjacent to white matter bundles.

We next sought to leverage a next-generation version of the scanner (v2), where hardware modifications improved resolution and signal uniformity, in addition to incorporating MD trace and ADC maps. For this study, the focus of the v2 scanner was on imaging patients with AIS. The v2 scanner was able to detect ischemic lesions as small as 0.15 mL (2.8 mm), achieve a sensitivity and specificity of >100% when detecting lesions >1.0 mL, improve signal uniformity in the white matter tracts, and achieve a ≈30% reduction in scan time. Together with the wider bore, this expedites workflows and improves integration of LF-MRI into clinical care.

There are limitations to our study. Although we evaluated different pulse sequences across both hardware versions, the number of AIS and stroke mimic cases was not evenly distributed across sequence-hardware combinations, and AIS cases were overrepresented relative to stroke mimics. Notably, only 1 stroke mimic was scanned on v2 hardware. However, the primary goal of this study was to improve the lower limit of detection for small ischemic strokes, which led to a focus on enrolling patients with AIS for the v2 hardware system.

Nevertheless, there remains a limit to the detection of tiny ischemic lesions on LF-MRI. While this did not alter the negative predictive value and positive predictive value for differentiating AIS from stroke mimics, it is possible that in the setting of transient ischemic attack, very subtle ischemic lesions may be missed. Future work should explore this possibility, including potential impacts on risk stratification and emergency department–based workflows. In addition, although we achieved a substantial reduction in acquisition duration with high scan tolerance and ease of operation, the scan time for LF-MRI sequences is generally longer than 1.5 and 3 T MRI counterparts. Evaluation of the sensitivity of different *b* values to AIS lesions may enable further reductions in scan time in the high-power gradient amplifier regime of the v2 system, which remains an avenue for future investigation. Despite the utility of LF-MRI for stroke triage, perfusion-weighted imaging and magnetic resonance angiography have not yet been developed at LF, currently precluding penumbral imaging and large vessel occlusion detection. Finally, although our study was conducted across multiple sites, limitation to major metropolitan hospitals may reduce generalizability, and acquisition of images on each system was based on chronological availability. A head-to-head clinical trial is needed to comprehensively compare the 2 hardware generations, ideally incorporating imaging within the same patient.

## Conclusions

We demonstrate optimization strategies at low magnetic field that improve diagnostic accuracy of DWI and signal uniformity, reduce scan time, and enable the detection of stroke lesions as small as 0.15 mL. These advancements highlight the potential role of portable LF-MRI as a diagnostic tool for AIS. The utility in clinical workflows may be beneficial in evaluating patients with suspected mild stroke, transient ischemic attack, or those who present with wake-up stroke. This technology may also be useful as a diagnostic tool for locations that do not otherwise have ready access to an MRI.

## ARTICLE INFORMATION

### Author Contributions

Dr Sorby-Adams, Dr Pinter, Dr Jaikumar, B.A. Parry, J. Pitts, Dr Lee, Dr Muir, Dr Nimjee, Dr Siddiqui, Dr de Havenon, Dr Sheth, Dr Goldstein, and Dr Kimberly contributed to the study design. Drs Sorby-Adams, Kirsch, O’Halloran, Keenan, and Iglesias contributed to sequence development. Dr Sorby-Adams, Dr Pinter, A. Demopoulos, Dr Jaikumar, O.K. Nelson, J. Guo, H. Brigger, I. Johnson, Dr Lee, and Dr Goldstein contributed to data acquisition. Dr Sorby-Adams, Dr Pinter, A. Demopoulos, Dr Bacchi, and Dr Kimberly analyzed the data. Dr Sorby-Adams, A. Demopoulos, and Dr Kimberly contributed to data interpretation and the initial draft of the manuscript. Dr Pinter, A. Demopoulos, Dr Kirsch, Dr Jaikumar, O.K. Nelson, Dr Bacchi, J. Guo, B.A. Parry, H. Brigger, I. Johnson, Dr de Havenon, Dr Sze, Dr O’Halloran, J. Pitts, Dr Lee, Dr Muir, Dr Nimjee, Dr Siddiqui, Dr Keenan, Dr Rosen, Dr Iglesias, Dr Sheth, and Dr Goldstein contributed to critical revision of the manuscript.

### Sources of Funding

This work was supported by the National Institutes of Health (NIH; EB031114, Drs Kimberly, Iglesias, Rosen, and Sheth), the Fulbright Commission (Drs Sorby-Adams and Bacchi), and the American Heart Association–Tedy’s Team postdoctoral fellowship award (Dr Sorby-Adams). Dr Iglesias is funded by NIH BRAIN Initiative (RF1 MH123195, UM1 MH130981) and NIH grants (R01 AG07098, RF1 AG080371, 1R21NS138995). Dr Rosen acknowledges the gracious support of the Kiyomi and Ed Baird MGH Research Scholar Award. Dr Keenan acknowledges funding from National Institute of Standards and Technology (https://ror.org/05xpvk416). All other coauthors report no relevant funding.

### Disclosures

Hyperfine provided the low-field magnetic resonance imaging scanners as part of sponsored research agreements (Drs Sheth, Siddiqui, and Kimberly). The company was not involved in the design of this investigator-initiated study or the publication decision. Dr Rosen is a founder and equity holder of Hyperfine, Inc and is also an equity holder of DeepSpin, GmbH. Dr Goldstein has performed consulting work for AstraZeneca, CSL Behring, Octapharma, Takeda, Pfizer, and Cayuga, all outside the scope of this project. Dr Keenan acknowledges assistance from Hyperfine through a Cooperative Research and Development Agreement. Certain commercial equipment, instruments, software, or materials are identified in this article to specify the experimental procedure adequately. Such identification is not intended to imply recommendation or endorsement by National Institute of Standards and Technology, nor is it intended to imply that the materials or equipment identified are necessarily the best available for the purpose. Dr Nimjee serves on the editorial board of *Stroke: Vascular and Interventional Neurology*. Editorial board members are not involved in the handling or final disposition of submissions. The other authors report no conflicts.

### Supplemental Material

Table S1

STROBE Checklist

## Supplementary Material


