# Supplementary material for: Enhanced Detection of Acute Ischemic Stroke With Low-Field MRI
Source: Stroke Vasc Interv Neurol. 2026 Jan 21;6(2):e002110. doi: 10.1161/SVIN.125.002110 (PMC12959426; doi:10.1161/SVIN.125.002110)
Supplement: Supplementary file 1 [file svi2-6-e002110-s001.pdf]

SUPPLEMENTARY MATERIAL

Enhanced Detection of Acute Ischemic Stroke with Low-Field MRI

Annabel Sorby-Adams PhD[1], Nandor K. Pinter MD[2,3], Amelia Demopoulos BA[1], John Kirsch PhD[4], Vinay Jaikumar MD[3], Olivia K. Nelson BS[5], Stephen Bacchi MBBS PhD[1], Jennifer Guo BS[1], Blair A. Parry BA[5], Hailey Brigger BA[6], Ian Johnson BA[6], Adam de Havenon MD MSCI[6], Gordon Sze MD[7], Rafael O'Halloran, PhD[8], John Pitts BS[8], Vivien H. Lee MD[9], Keith W. Muir MB ChB MD[10], Shahid M. Nimjee MD PhD[9], Adnan Siddiqui MD PhD[3], Kathryn E. Keenan PhD[11], Matthew S. Rosen PhD[4], Juan Eugenio Iglesias PhD[4,12,13], Kevin N. Sheth MD[6], Joshua N. Goldstein MD PhD[5], W. Taylor Kimberly MD PhD[1]

[1] Department of Neurology and the Center for Genomic Medicine, Massachusetts General Hospital and Harvard Medical School, Boston, Massachusetts, USA
[2] Department of Radiology, Jacobs School of Medicine & Biomedical Sciences, State University of New York at Buffalo, Buffalo, New York, USA
[3] Department of Neurosurgery, Jacobs School of Medicine & Biomedical Sciences, State University of New York at Buffalo, Buffalo, New York, USA
[4] Athinoula A. Martinos Center for Biomedical Imaging, Massachusetts General Hospital and Harvard Medical School, Charlestown, Massachusetts, USA
[5] Department of Emergency Medicine, Massachusetts General Hospital and Harvard Medical School, Boston, Massachusetts, USA
[6] Department of Neurology, Center for Brain & Mind Health, Yale New Haven Hospital and Yale School of Medicine, New Haven, CT, USA
[7] Division of Neuroradiology, Department of Radiology and Biomedical Imaging, Yale New Haven Hospital and Yale University School of Medicine, New Haven, CT, USA
[8] Hyperfine Incorporated, Guilford, Connecticut, USA
[9] The Ohio State University Wexner Medical Center, Columbus, Ohio, USA
[10] School of Psychology and Neuroscience, University of Glasgow, Glasgow, UK
[11] National Institute of Standards and Technology, Boulder, Colorado, USA
[12] Hawkes Institute, University College London, London, UK
[13] Computer Science and Artificial Intelligence Laboratory, Massachusetts Institute of Technology, Cambridge, Massachusetts, USA

Correspondence
W. Taylor Kimberly, MD, PhD
55 Fruit Street, Lunder 644
Boston, MA 02114 USA
wtkimberly@mgh.harvard.edu
Phone: 857-238-5644

Supplementary Table 1. Hardware specifications for Hyperfine Inc. v1 and v2 devices

	v1	v2
Magnet		
Field strength (mT)	64	64
Type	Permanent	Permanent
Cryogens	None	None
Physical specifications		
Geometry	C-arm	H-arm
Power requirements	15 A, 120 V	15 A, 120 V
Weight (kg)	606kg	631kg
Dimensions (cm)		
Height	150	144
Width	81	81.4
Depth	97	144
Bore size (cm)		
Height	31.5	34
Width	61	91.5
Patient transfer bridge		
Supported weight (max, kg)	200	200
Width of bridge (cm)	56	67
Spatial Homogeneity (ppm)[a]	1111	1111
Gradient system		
Maximum gradient amplitude (mT/m)		
x	24.3	33.9
y	22.9	33.2
z	38.5	66.2
Slew rate (T/m/s)		
x	24	18.8
y	22	18.4
z	21	13.0
Cooling system	Passive	Forced air
Head coil		
Type	Transmit / receive	Transmit / receive
Receiver coil design	8-channel	8-channel
Transmit coil design	1-channel	1-channel

[a] Homogeneity over 200 mm diameter spherical volume
